# Spatial Memory and Microglia Activation in a Mouse Model of Chronic Neuroinflammation and the Anti-inflammatory Effects of Apigenin

**DOI:** 10.3389/fnins.2021.699329

**Published:** 2021-07-30

**Authors:** Rose Chesworth, Rashmi Gamage, Faheem Ullah, Sandra Sonego, Christopher Millington, Amanda Fernandez, Huazheng Liang, Tim Karl, Gerald Münch, Garry Niedermayer, Erika Gyengesi

**Affiliations:** ^1^School of Medicine, Western Sydney University, Campbelltown, NSW, Australia; ^2^Department of Pharmacology, School of Medicine, Western Sydney University, Penrith, NSW, Australia; ^3^Translational Neuroscience Lab, Center for Translational Science, Department of Environmental Sciences, Robert Stempel College of Public Health, Florida International University, Port St. Lucie, FL, United States; ^4^Department of Neurology, Translational Research Institute of Brain and Brain-Like Intelligence, Shanghai Fourth People’s Hospital Affiliated to Tongji University School of Medicine, Shanghai, China; ^5^Neuroscience Research Australia, Sydney, NSW, Australia; ^6^School of Medical Sciences, University of New South Wales, Sydney, NSW, Australia; ^7^School of Science, Western Sydney University, Penrith, NSW, Australia

**Keywords:** microglia, aging, flavonoids, Alzheimer’s disease, spatial memory, animal – mouse, stereological analyses

## Abstract

Chronic neuroinflammation characterized by microglia reactivity is one of the main underlying processes in the initiation and progression of neurodegenerative diseases such as Alzheimer’s disease. This project characterized spatial memory during healthy aging and prolonged neuroinflammation in the chronic neuroinflammatory model, glial fibrillary acidic protein-interleukin 6 (GFAP-IL6). We investigated whether chronic treatment with the natural flavonoid, apigenin, could reduce microglia activation in the hippocampus and improve spatial memory. GFAP-IL6 transgenic and wild-type-like mice were fed with apigenin-enriched or control chow from 4 months of age and tested for spatial memory function at 6 and 22 months using the Barnes maze. Brain tissue was collected at 22 months to assess microgliosis and morphology using immunohistochemistry, stereology, and 3D single cell reconstruction. GFAP-IL6 mice showed age-dependent loss of spatial memory recall compared with wild-type-like mice. Chronic apigenin treatment decreased the number of Iba-1^+^ microglia in the hippocampus of GFAP-IL6 mice and changed microglial morphology. Apigenin did not reverse spatial memory recall impairment in GFAP-IL6 mice at 22 months of age. GFAP-IL6 mice may represent a suitable model for age-related neurodegenerative disease. Chronic apigenin supplementation significantly reduced microglia activation, but this did not correspond with spatial memory improvement in the Barnes Maze.

## Introduction

Neuroinflammation is a fundamental process underlying the initiation and progression of neurodegenerative diseases such as Alzheimer’s disease (AD). It is characterized by microglial activation, reactive astrogliosis, expression of pro-inflammatory cytokines, and the release of reactive oxygen and nitrogen species. Although immune activation in the central nervous system (CNS) occurs as a protective reaction against microbial infection, acute injury or disease, uncontrolled and sustained neuroinflammation can create a neurotoxic environment that leads to neuronal injury, synaptic loss, and neuronal death. Importantly, while low level neuroinflammation is evident during healthy aging (Calder et al., 2017), this appears to be exacerbated in neurodegenerative diseases such as AD (Seabrook et al., 2006; Sochocka et al., 2017), and may lead to cognitive decline, behavioral changes and neurodegeneration evident in these disorders.

Aging is the main cause of microglial activation caused by an innate inflammatory response specific to the CNS (Frank-Cannon et al., 2009; Tohidpour et al., 2017). Microglia, the primary innate immune cells of the brain, function as immune sentinels and effectors to synaptic pruning, as well as modulators of higher cognitive function (e.g., learning and memory) (Bazan et al., 2012; Ginhoux et al., 2013; DiSabato et al., 2016). Microglia also have macrophage-like activities and contribute to the maintenance of CNS homeostasis (Bazan et al., 2012; Ginhoux et al., 2013; DiSabato et al., 2016). Morphologically, they can be categorized into ramified (resting), primed, reactive, and amoeboid (phagocytic), and each category shows differences in cell body morphology, branching structure and process length (Soltys et al., 2001; Stence et al., 2001; Karperien et al., 2013; Torres-Platas et al., 2014). Ionized calcium binding adaptor molecule 1 (Iba-1) is an actin binding protein restricted to microglia in the CNS, and is upregulated in reactive microglia following brain injury and disease (Ohsawa et al., 2004; Hovens et al., 2014). Microglial function during aging is marked by a switch from their neuroprotective inflammatory nature which involves the production of anti-inflammatory mediators, to a proinflammatory profile in the aged brain upon activation (Sochocka et al., 2017). These features suggest that aging has a significant impact on microglial function and subsequent neurotoxicity.

Despite decades of intensive research into neurodegenerative disorders, current pharmacological treatment is focused on symptom management, and no treatments limit disease progression or prevent disease occurrence (Goldman et al., 2018). There is a pressing need to develop novel therapies which can halt or reverse underlying neuroinflammatory and neurodegenerative processes.

There has been a growing interest in the use of natural polyphenols as multipotent agents to combat neurological disease. Epidemiological evidence demonstrates that dietary flavonoid intake is associated with a decreased risk of neurodegenerative diseases (Bhullar and Rupasinghe, 2013). Furthermore, animal and clinical studies indicate dietary flavonoids possess neuroprotective properties, defending neurons against oxidative stress, attenuating neuroinflammation and improving cognition and learning (Vauzour et al., 2008; Spencer et al., 2012).

Apigenin (4′,5,7-trihydroxyflavone) is a natural flavonoid of low molecular weight (MW 270.24), which is found in a variety of plants, fruits and vegetables, and has neuroprotective potential (Patel et al., 2007). Apigenin shows potent antimicrobial, anti-inflammatory, antioxidant, anti-depressive-like, and antitumorigenic properties (Viola et al., 1995). It exerts many of its effects through interactions with the signaling molecules in the three major mitogen-activated protein kinase pathways [extracellular signal-regulated kinase (ERK), c-Jun N-terminal kinase, and p38] in murine and human cell culture models (Viola et al., 1995; Patel et al., 2007). Apigenin is considered safe with low levels of toxicity, and appears to cross the blood brain barrier (BBB) (Yang et al., 2014).

The anti-inflammatory properties of apigenin are evident through a dose-dependent suppression of the inflammatory mediators nitric oxide (NO) and prostaglandin, *via* inhibition of inducible NO synthase and cyclooxygenase-2 (Cox-2) in BV-2 murine microglial cells (Rezai-Zadeh et al., 2008). Apigenin also strongly suppresses levels of Cluster of differentiation 40 (CD40), tumor necrosis factor α (TNF-α) and interleukin-6 (IL-6) *via* inhibition of interferon-γ (IFN-γ)-induced phosphorylation of signal transducer and activator of transcription 1 in murine microglia (Elsisi et al., 2005).

Cognitive enhancing effects have also been reported following apigenin treatment. Acute apigenin administration improves long term memory in a passive avoidance task in male Wistar rats (Popovic et al., 2014). Chronic apigenin also reduces Morris Water Maze (MWM) cognitive impairment in rodent models of diabetes (Mao et al., 2015) and isoflurane-induced cognitive dysfunction (Chen et al., 2017). Importantly, neuroprotective effects of chronic apigenin supplementation have been shown in the amyloid precursor protein/presenilin 1 (*APPxPS1*) AD mouse model (Zhao et al., 2013). Three months of chronic apigenin treatment improved learning and memory in the MWM in *APPxPS1* mice, and reduced fibrillar amyloid deposits and lowered insoluble β-amyloid concentrations (Zhao et al., 2013). These findings suggest that apigenin treatment can restore cognition in various preclinical models; however, these models do not necessarily elicit a strong neuroinflammatory phenotype, and the treatment potential of apigenin in a model of chronic neuroinflammation is unclear. This study aimed to determine whether the anti-inflammatory effects of apigenin treatment are the mechanism by which cognitive deficits are ameliorated.

In the present study, we utilized the glial fibrillary acidic protein-interleukin 6 (GFAP-IL6) transgenic mouse as a model of sustained microglia activation (Campbell et al., 1993). In this model, inflammation is localized in the brain by triggering chronic expression of the cytokine IL-6 *via* the GFAP astrocytic promoter (Campbell et al., 1993). GFAP-IL6 mice exhibit fundamental components of progressive neurodegeneration including neuronal loss and atrophy, chronic activated microglia and astrocytes, increased expression of inflammatory mediators (Gyengesi et al., 2019), breached BBB, and age-dependent motor and cognitive impairment (Campbell et al., 1993; Gyengesi et al., 2019; Ullah et al., 2020a, b). We chose to further characterize the GFAP-IL6 mouse model so that the therapeutic effect of anti-inflammatory drugs on cognition could be tested. The aim of this study was to investigate age-related cognitive decline and microglial reactivity described by morphological changes in GFAP-IL6 mice, and to determine if chronic apigenin treatment could ameliorate cognitive decline and microglial activation in both GFAP-IL6 mice and age-matched controls. These experiments were designed to determine if apigenin may be an appropriate novel treatment for cognitive decline evident in neurodegenerative diseases characterized by elevated neuroinflammation.

## Materials and Methods

### Animals

Male and female WT (C57BL/6) and GFAP-IL6 heterozygous mice (*n* = 6–12/genotype and treatment group) were housed in the animal facility of the School of Medicine, Western Sydney University under a temperature-controlled environment (23°C and 60 ± 10% humidity), with a 12/12 h light–dark cycle. Mice were kindly donated by Prof Iain Campbell (University of Sydney) (Campbell et al., 1993). Mice were provided with food and water *ad libitum*. Mice had access to nesting material and tissues, as well as paper and PVC rolls for environmental enrichment. Four-month-old GFAP-IL6 and their non-transgenic WT littermates were used. All experimental procedures were approved by the Western Sydney University Animal Care and Ethics Committee (approval ID: A11393) and carried out in accordance with the Australian Code of Practice for the Care and Use of Animals for Scientific Purposes.

### Grouping of Animals and Apigenin Feeding

Animals were randomly assigned to four groups: WT and GFAP-IL6 mice fed with control food pellets (i.e., standard mouse chow, Specialty Feeds, Perth, Australia), and WT and GFAP-IL6 mice fed with apigenin containing pellets (400 ppm apigenin). Apigenin was supplied by Nutrafur S.A. (Alcantarilla, Murcia, Spain). Apigenin was mixed with powdered standard mouse chow (Specialty Feeds), and 15% water was added before the mixed diet was introduced to the pellet machine. In the pelleting machine, the temperature of the product did not exceed 43°C. After pelleting, the moisture content was reduced to stabilize the diet against microbial storage damage. This was done by loading the pellets into a shallow tray in an air dryer set at 45°C for 3 h. Over the drying cycle, the temperature of the pellets was increased slowly from 25 to 45°C. Mouse chow pellets were stored at room temperature in vacuum packed bags in the animal housing facility at the School of Medicine, Western Sydney University. Starting from 4 months of age, mice were fed with control chow or apigenin-containing chow and feeding continued until the end of the experiment. Food intake and body weight of the mice were recorded initially to determine tolerance to chow and dosage. The actual average daily Apigenin dosing of the mice (mg/kg bodyweight per day) was calculated to be 110 mg/kg. Behavioral testing was conducted at 6 and 22 months of age; mice were perfused for histology after testing at 22 months.

### Behavioral Testing

The Barnes Maze (BM) is a navigational maze used to assess spatial learning and memory (Gawel et al., 2019). The BM was a plastic circular platform 91 cm in diameter and 90 cm above the floor (Stoelting, Wood Dale, IL, United States). There were 20 evenly spaced holes 5 cm apart around the perimeter of the maze; 19 of which were false holes, and one of which was an escape hole. Intra-maze cues (i.e., cards containing a different shape) were placed 5 cm from the maze at every 5th hole, and extra-maze cues (e.g., room furniture) were kept consistent during acquisition and probe to facilitate spatial navigation. Aversive stimuli (i.e., bright lights of 950 lux, 85 dB binaural beats) were used to motivate mice to find the escape hole during all trials. Between each trial, the apparatus was cleaned thoroughly with 70% ethanol and allowed to fully dry.

Test mice were trained to find the escape hole over 3 days. On the first day (habituation), mice were given one 20 s trial to explore the apparatus. At the end of the trial, the experimenter gently guided the mouse to the escape hole, and the mouse was left undisturbed in the escape hole with the lights and sound turned off for 2 min. During task acquisition (next 2 days), mice were given three 180 s trials daily separated by a 20–30 min intertrial interval, in which mice needed to find the escape hole. If the mouse did not find the escape hole within the trial, it was gently guided to the escape hole at the end of the trial. Once the mouse entered the escape hole, the lights and sound were switched off, and the mouse left undisturbed for 2 min on the first Acquisition day, and for 1 min on the second Acquisition day. Data for acquisition are presented as the average of the three trials per day.

There was a 72-hour rest period between Acquisition and the Probe trial. During the single Probe trial, the escape hole was replaced with a false hole, and the mouse was allowed to explore the apparatus for 90 s.

Video footage was collected and analyzed with ANY-maze (Stoelting, Wood Dale, IL, United States) v.4.99. BM performance during acquisition was assess *via* primary latency (latency until first visit to the escape hole), path length (distance traveled to enter escape hole), errors (entries into false holes), and speed. At Probe, attempted entries into the escape hole was compared to the average of entries into all other holes, as well as total distance traveled at Probe.

### Tissue Preparation and Immunohistochemistry

For histological analysis [methods (Gyengesi et al., 2019)], tissue samples were prepared from all experimental cohorts. Mice were anesthetized with pentobarbitone (30–50 mg/kg i.p.) and were transcardially perfused with 30 ml of 0.9% normal saline using a peristaltic pump, followed by 60 ml of 4% cold paraformaldehyde (Merck) in 0.1M phosphate saline buffer (PBS) solution. Brains were collected and post-fixed in 4% paraformaldehyde for 24 h at 4°C, and then transferred to 30% sucrose in 0.1M PBS solution for cryoprotection. After the brains sank to the bottom of the container, they were embedded and frozen in 6% gelatin in 0.1M PBS. Forty μm thick coronal sections were cut in eight series using a Leica CM 1950 cryostat.

Immunohistochemistry assays were performed on every sixth section (Gyengesi et al., 2019). All washing and incubation procedures were performed using 0.1 M PBS. Sections were incubated in 2% normal goat serum (Thermo Fisher Scientific, #31872) with 0.01% Triton-X, followed by a washing step. Then sections were incubated in primary rabbit-anti-Iba-1 antibody (1:500, Wako, # 019-19741) with 0.01% Triton-X for 2 days at 4°C, followed by the fluorescent secondary antibody (1:200, goat anti-rabbit IgG, Alexa 488; Thermo Fisher Scientific, #1853312) for 2 h at room temperature. The sections were washed, mounted, and coverslipped with a fluorescent mounting medium (Vector Laboratories, #H1400).

### Stereological Counting

The estimated number of Iba-1^+^ microglial cells in the hippocampus was counted using the Zeiss AxioImager M2 microscope equipped with MBF Biosciences StereoInvestigator (Bastide et al., 2014). The contour of the hippocampal formation was first drawn under the 2.5× objective. The size of the counting frame for the WT mice was 120 × 120 μm; the size of the counting frame for the GFAP-IL6 mice was 60 × 60 μm; and the counting grid was 800 × 800 μm, for all of the cohorts. The guard zone was 1 μm at the top and the bottom of the sections. Microglia were plotted on the screen using a marker as the focus, which moved from the top to the bottom of the sections using a 63× oil objective. This led to a Gunderson coefficient error of less than 0.1 in all cases (*m* = 1).

### Three-Dimensional Reconstruction of Hippocampal Microglia

Cover slipped samples were prepared as detailed above and imaged using a Confocal ZEISS Laser Scanning Microscope (LSM800) with an argon laser and processed using the Zen Blue^®^ software package. The super resolution images were obtained using Airyscan with 1.3× crop area, and Z-stacks were captured using a 63× objective and NA1.0 for reflective imaging, at a step size of 0.18–2 μm (unless otherwise specified). Reflective imaging was achieved using the 561 nm wavelength. The 3D reconstruction of microglia was performed using Neurolucida 360^®^ (MBF Bioscience) software. Extended depth of focus images were obtained by collapsing the Z-stacked 3D images of resolution 1596 × 1596 pixels, to better identify the objects and provide greater accuracy. After importing the Z-stacks to Neurolucida 360 software, microglia were manually reconstructed using the user-guided mode, along the required planes, obtaining a reconstructed 3D image of each cell [methods (Gyengesi et al., 2019)].

### Analysis of Reconstructed Microglia

Morphometric data of each microglial cell was extracted with the Neurolucida 360^®^ built-in analysis software, Neurolucida Explorer, and each reconstructed cell was subject to multiple measures. As described recently by our group, the soma area, μm^2^; soma perimeter, μm; convex two-dimensional (2D) area of the branching field, μm^2^; convex perimeter of the branching field, μm; the total length of all processes, μm; the total number of processes from the soma; and the number of nodes (branch points) were measured using Branched Structure and Convex Hull analysis (Ullah et al., 2020b, a). Sholl analysis for each microglial cell was performed to determine changes cell size in relation to the distance from the cell soma (Ullah et al., 2020b, a). This analysis was performed in nested concentric spheres (radius, *r*) centered at the cell soma, *r* = 0, with increase in size by a constant change in radius of 5 μm steps and counting the number of compartments crossing a given radius. Sholl diagrams were averaged and maximum value was obtained as the maximum number of crossings. This analysis determined the number of intersections, the process length (μm), the surface area of the cells (μm^2^), the process volume (μm^3^), the process diameter (μm), and a number of nodes of the cells for each radius.

### Data Analysis

All behavioral and histological data was collapsed across sex, as we have not detected sex effects in GFAP-IL6 mice previously (Gyengesi et al., 2019). Behavioral data was analyzed in SPSS (IBM, v.25) with three- and four-way repeated measures ANOVA with the repeated factors “days” (acquisition days 1/2) or “location” (escape hole/other holes) and “age” (6/22 months), and the between factors “genotype” (WT/GFAP-IL6) and “diet” (control/Apigenin). Bonferroni *post hoc* tests were performed when significant interactions were detected. To assess the percentage of escape hole entries, data was split by “genotype,” “age” and “diet,” and single sample *t*-tests performed (test value: 5%, i.e., 1/20 is chance level).

The distinct morphological features of hippocampal microglia were compared using two-way ANOVA with between factors “genotype” and “diet,” followed by Bonferroni *post hoc* tests in Prism GraphPad (version 8). Sholl analysis data was compared using three-way ANOVA with the factors “genotype,” “diet,” and “radius.” Correlations of the Convex Hull area with the distinct morphological features of the microglia were analyzed using linear regression (*R*^2^) and correlation tests in Prism GraphPad (version 8). All behavioral and morphological data are presented as mean ± SEM.

## Results

### GFAP-IL6 Mice Exhibit Age-Dependent Cognitive Impairment, Which Was Not Improved by Chronic Apigenin Treatment

To investigate how the accumulating effects of aging and chronic microglial activation impact on cognitive function and if cognitive decline could be ameliorated by apigenin supplementation, we performed the BM test on apigenin and standard diet-fed WT and GFAP-IL6 mice at 6 and 22 months of age. Repeated measures ANOVA revealed no significant effect of “diet” on the body weight of mice across a 4-month period (Supplementary Figure 1).

#### Higher Primary Latency During Acquisition in 22 Months Old GFAP-IL6 Mice

**FIGURE 1:**
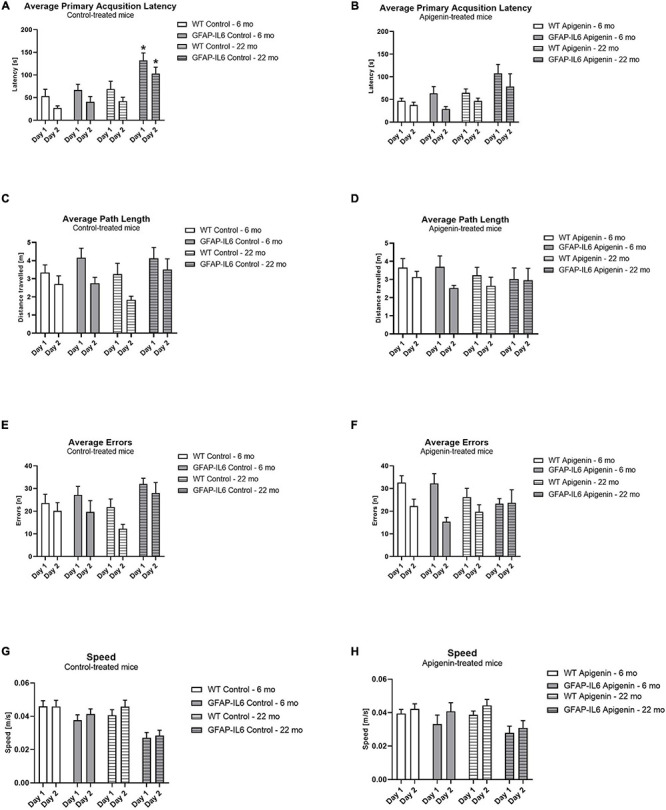
**(A–H)** Effect of age and apigenin diet on Barnes Maze Acquisition in WT and GFAP-IL6 mice. Primary Acquisition latency (s) is presented for standard diet-fed mice in **(A)** and apigenin-fed mice in **(B)**, and Average Path length (m) is shown for standard diet-fed mice in **(C)** and apigenin-fed mice in **(D)**. Average Errors (n) is displayed for standard diet-fed mice in **(E)** and apigenin-fed mice in **(F)**, while average speed (m/s) is presented for standard diet-fed mice in **(G)** and apigenin-fed mice in **(H)**. Data analyzed with four-way ANOVA, followed by Bonferroni *post hoc* tests. *Post hoc* effects of “genotype” within the same age and treatment group are indicated by stars (*p < 0.05). Data presented as means ± SEM.

Glial fibrillary acidic protein-interleukin 6 mice exhibited a higher primary latency compared to WT controls [“genotype” *F*(1,34) = 15.5, *p* < 0.0001]; this was evident at 22 months, but not at 6 months [“age” × “genotype” *F*(1,34) = 6.0, *p* = 0.02] (Figures 1A,B). In both genotypes, primary latency decreased across the 2 days of training, indicating task acquisition [“days” *F*(1,34) = 44.6, *p* < 0.0001; no interaction with “genotype” *p* > 0.05], and primary latency increased with age [“age” *F*(1,34) = 17.1, *p* < 0.0001]. Apigenin treatment did not modify primary latency in either genotype or age group [“diet” *F*(1,34) = 1.0, *p* = 0.3; no interactions with “diet”]. Together, this indicates a higher primary latency in GFAP-IL6 months at 22 months only; and this was not ameliorated by apigenin treatment.

#### No Effect of Age or Apigenin Treatment on Path Length During Acquisition

During acquisition, one path length data point for a WT apigenin-fed mouse was lost due to a technical error.

Average path length decreased across the 2 days of learning in all groups when data was averaged across all groups, suggesting improvement in learning over time [“days” *F*(1,33) = 11.8, *p* < 0.0001] (Figures 1C,D). Note that while path length did not appear to decrease across days in GFAP-IL6 22 month old mice (Figures 1C,D), this was not sufficient to alter the mean path length for all other groups (no interactions between “days” and “genotype” or “diet” or “age,” all *p*’s > 0.05). Path length was not different between the two ages [“age” *F*(1,33) = 0.5, *p* = 0.5] or genotypes [“genotype” *F*(1,33) = 1.7, *p* = 0.2]. Apigenin treatment did not affect path length [“diet” *F*(1,33) = 0.1, *p* = 0.7; no interaction with “diet” *p* > 0.05]. Together, this suggests that on average both genotypes and age groups decreased their path length across acquisition, and apigenin treatment had no effect on path length.

#### No Effect of Age or Apigenin Treatment on Errors Made During Acquisition

Age did not increase errors in the BM [“age” *F*(1,34) = 0.1, *p* = 0.7; no interactions with “age” *p* > 0.05]. All groups decreased their errors across the 2 days of acquisition, indicating learning of the task [“days” *F*(1,34) = 22.8, *p* < 0.0001; no interactions between “days” and “genotype” or “diet,” *p*’s > 0.05] (Figures 1E,F). GFAP-IL6 mice did not make more errors than WT mice at either age [“genotype” *F*(1,34) = 1.6, *p* = 0.2; no interactions with “genotype” *p* > 0.05]. Apigenin treatment did not affect errors in either genotype or age group [“diet” *F*(1,34) = 0.4, *p* = 0.6; no interactions with “diet” *p* > 0.05]. This suggests that both genotypes and ages decreased their errors across acquisition, and apigenin treatment had no effect on errors.

#### GFAP-IL6 Mice Were Slower Than WTs Mice During Acquisition, Particularly at 22 Months Old

Overall, GFAP-IL6 mice were slower than WT mice [“genotype” *F*(1,34) = 19.2, *p* < 0.0001] (Figures 1G,H). Older mice were slower than younger mice [“age” *F*(1,34) = 5.8, *p* = 0.02], and an “age” × “genotype” interaction trend [*F*(1,34) = 3.8, *p* = 0.06] suggests GFAP-IL6 mice tended to be slower at 22 months than at 6 months, where this change was not evident in WT mice. Mean speed increased across testing days in all groups [“days” *F*(1,34) = 7.0, *p* = 0.01; no interactions between “days” and “genotype” or “diet” or “age,” all *p*’s > 0.05], but was unaffected by apigenin treatment [“diet” *F*(1,34) = 0.8, *p* = 0.4; no interactions between “diet” and “genotype” or “days” or “age,” all *p*’s > 0.05]. These results indicate GFAP-IL6 mice were slower than WT mice, and older mice were slower than younger mice.

#### Reduced Escape Hole Entries at Probe in 22 Months Old GFAP-IL6 Mice

**FIGURE 2:**
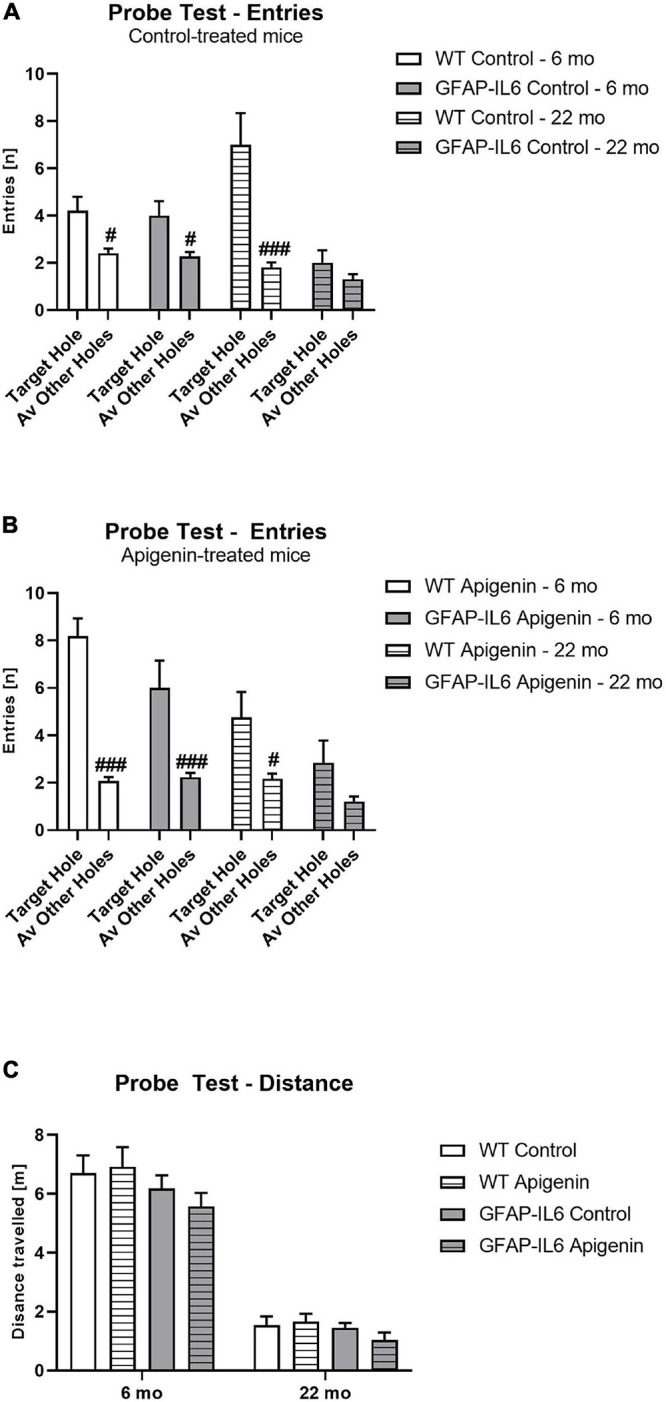
**(A–C)** Effect of age and apigenin diet on Barnes Maze Probe Test Performance in WT and GFAP-IL6 mice. Entries (n) into the escape hole is presented for **(A)** control and **(B)** apigenin-fed mice. Distance traveled (m) at Probe for all groups is presented in **(C)**. A main effect of genotype and age in **(C)** indicates reduced distance traveled in GFAP-IL6 mice compared to WT controls, and reduced distance traveled in 22-month-old mice compared to 6-month-old mice. Data analyzed with three- and four-way ANOVA, followed by Bonferroni *post hoc* tests. *Post hoc* effects of “location” within the same age and treatment group are indicated by hash symbols (#*p* < 0.05, ###*p* < 0.001). Data presented as means ± SEM.

Overall, all groups attempted to enter the escape hole more than other holes [“location” *F*(1,31) = 72.0, *p* < 0.0001]. GFAP-IL6 mice made fewer hole entries compared to WT mice [“genotype” *F*(1,31) = 14.7, *p* < 0.0001], and aged mice also made fewer hole entries compared to younger mice, irrespective of genotype or diet [“age” *F*(1,31) = 7.7, *p* = 0.009]. There was no effect of apigenin treatment on escape hole entries [“diet” *F*(1,31) = 2.7, *p* = 0.1]. A “location” × “genotype” interaction [*F*(1,31) = 7.7, *p* = 0.009] indicates GFAP-IL6 mice exhibited poorer discrimination than WT mice, irrespective of age or diet. An “age” × “genotype” interaction [*F*(1,31) = 6.1, *p* = 0.02] indicates GFAP-IL6 mice made fewer hole entries at 22 months than WT mice; this was not evident at 6 months. There was no “age” × “genotype” × “location” interaction (*p* > 0.05). *Post hoc* tests confirm that both genotypes entered the target hole more than other holes at 6 months, but this discrimination is only evident in WT mice at 22 months (Figures 2A,B).

#### Reduced Distance Traveled at Probe in GFAP-IL6 Mice and in 22 Months Old Mice

At Probe, GFAP-IL6 mice traveled less than WT controls [“genotype” *F*(1,31) = 8.0, *p* < 0.0001; no interactions with “age” or “diet” *p*’s > 0.05] (Figure 2C). Older mice traveled less than younger mice [“age” *F*(1,31) = 47.8, *p* < 0.0001; no interactions with “genotype” or “diet” *p*’s > 0.05]. Apigenin treatment did not affect distance traveled at probe [“diet” *F*(1,31) = 0.01, *p* = 0.9; no interactions with “age” or “genotype” *p*’s > 0.05].

#### Percentage of Escape Hole Entries at Probe Impaired in All Groups at 22 Months Except WT Standard Diet-Fed

Single sample *t*-tests comparing escape hole entries to chance level (i.e., 5% or 1/20 holes) indicate all groups entered the escape hole more than chance at 6 months [WT standard diet-fed: *t*(9) = 3.32, *p* = 0.009; GFAP-IL6 standard diet-fed: *t*(9) = 2.81, *p* = 0.02; WT apigenin-fed: *t*(8) = 4.35, *p* = 0.002; GFAP-Il6 apigenin-fed: *t*(5) = 2.97, *p* = 0.03]. However, at 22 months, only WT mice entered the escape hole more than chance [WT standard diet-fed: *t*(9) = 2.27, *p* = 0.049; GFAP-IL6 standard diet-fed: *t*(9) = 1.35, *p* = 0.2; WT apigenin-fed: *t*(11) = 2.24, *p* = 0.047; GFAP-Il6 apigenin-fed: *t*(9) = 1.85, *p* = 0.1].

### Apigenin Decreased the Number of Hippocampal Iba-1^+^ Microglia in GFAP-IL6 Mice

**FIGURE 3:**
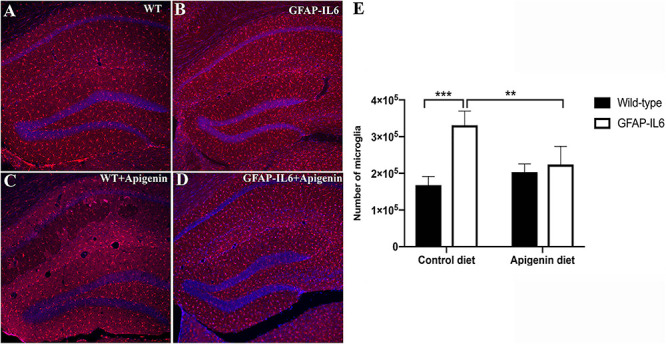
**(A–E)** Apigenin decreased the number of Iba-1^+^ microglia in the hippocampus of GFAP-IL6 mice at 22 months of age. **(A–D)** Representative photographs showing Iba-1^+^ microglia distribution in the hippocampus in **(A)** WT standard diet-fed mice, **(B)** GFAP-IL6 standard diet-fed mice, **(C)** WT apigenin-fed mice, and **(D)** GFAP-IL6 apigenin-fed mice. Scale bar represents 200 um. **(E)** Stereological estimation of total microglia counts in the hippocampus in 22-month-old WT and GFAP-IL6 mice fed control or apigenin-enriched diets. Data presented as mean ± SEM. *Post hoc* effects of “diet” indicated by asterisk, **p < 0.005, ***p < 0.001, *post hoc* effects of “genotype” indicated by hash symbols, ^###^*p* < 0.001.

To determine effects of apigenin on hippocampal microglial populations, immunohistochemistry and stereological counting of Iba-1^+^ microglia was performed in the hippocampus of control and apigenin-fed mice. Control GFAP-IL6 mice had 331,200 ± 19,400 Iba-1^+^ microglia, which was significantly higher than that of WT mice (244,100 ± 22,400) [“genotype” *F*(1,14) = 29.4, *p* < 0.001] (Figures 3A,B,E). A significant “diet” × “genotype” interaction showed that GFAP-IL6 apigenin-fed mice had lower microglia numbers than those on control diet [*F*(1,14) = 17.5, *p* < 0.001]. However, “diet” alone seemed to have a marginally significant effect on microglia numbers [*F*(1,14) = 4.46, *p* = 0.05]. *Post hoc* tests confirmed that apigenin significantly reduced the number of Iba-1^+^ microglia in the GFAP-IL6 animals by 32.2% to 224,104 ± 22,030 (*p* < 0.005); this was similar to levels in WT standard diet-fed levels (Figures 3C,D,E). Apigenin treatment in WT mice did not alter hippocampal microglia numbers (*p* = 0.21).

### Hippocampal Microglia Morphology Is Altered by the GFAP-IL6 Genotype

**FIGURE 4:**
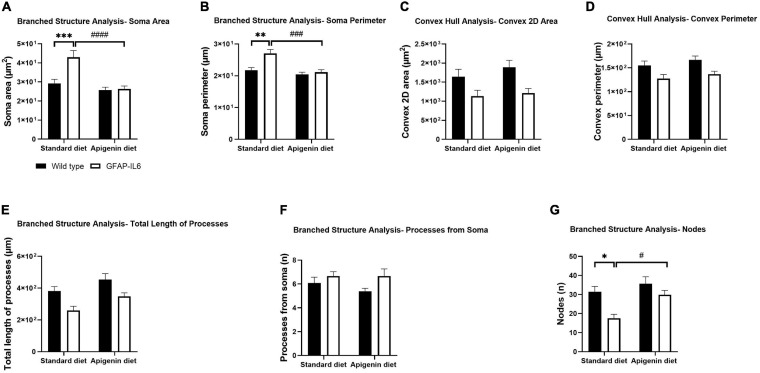
Effects of apigenin diet on Iba-1^+^ microglial cell morphology in the hippocampus. Quantitative analysis of the morphological changes in Iba-1^+^ microglia, including **(A)** soma area (μm^2^), **(B)** soma perimeter (μm), **(C)** convex 2D area (μm^2^), **(D)** convex perimeter (μm), **(E)** process length (μm), **(F)** number of processes from soma (n), and **(G)** number of nodes (n). Data presented as mean ± SEM and analyzed with two-way ANOVA followed by Bonferroni *post hoc* tests. *Post hoc* effects of “genotype” are represented in asterisks (^∗^*p* < 0.05, ^∗∗^*p* < 0.01, ^∗∗∗^*p* < 0.001, ****p < 0.0001); *post hoc* effects of “diet” are represented by hash symbols (^#^*p* < 0.05, ^##^*p* < 0.01, ^###^*p* < 0.001, ^####^*p* < 0.0001).

**FIGURE 5:**
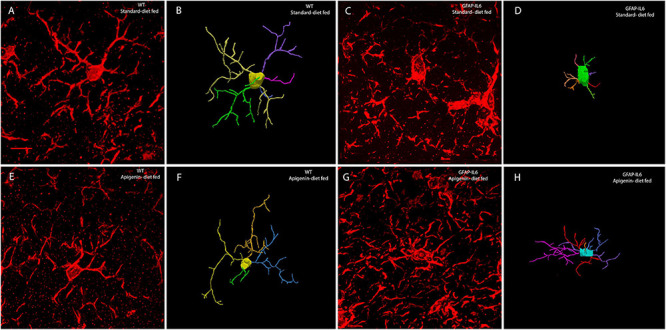
Representative confocal images of reconstructed microglia from WT standard diet-fed **(A,B)**, GFAP-IL6 standard diet-fed **(C,D)**, WT apigenin-fed **(E,F)**, and GFAP-IL6 apigenin-fed **(G,H)** cohorts. The extended depth of focus images obtained from collapsing 3D confocal microscopy images of Iba-1^+^ microglia obtained under 63× oil immersion objective, along with the corresponding manually reconstructed images in Neurolucida 360. GFAP-IL6 mice (apigenin diet) **(G,H)** had a significantly reduced soma area, soma perimeter and a larger number of nodes than that of GFAP-IL6 mice (standard diet) **(C,D)**. (Scale bar 10 μm).

To investigate Iba-1^+^ hippocampal microglial morphology, we used branched structure analysis with “diet” and “genotype” as variables. A main effect of “genotype” was observed in the measurements of soma area [“genotype” *F*(1,56) = 9.814, *p* = 0.0028], soma perimeter [“genotype” *F*(1,56) = 10.84, *p* = 0.0017] and number of nodes [“genotype” *F*(1,56) = 11.08, *p* = 0.0015], with control GFAP-IL6 mice exhibiting larger soma area (42.98 ± 3.454 μm^2^), soma perimeter (27.04 ± 1.216 μm) and lower number of nodes (17.53 ± 2.131) compared to control WT mice [soma area (29.26 ± 2.130 μm^2^), soma perimeter (21.72 ± 0.8491 μm) and nodes (31.42 ± 2.805)] (Figures 4, 5, and Supplementary Table 1).

A main effect of “diet” was observed in the measurements of soma area [“diet” *F*(1,56) = 19.40, *p* < 0.0001], soma perimeter [“diet” *F*(1,56) = 15.57, *p* = 0.0002], and number of nodes [“diet” *F*(1,56) = 7.868, *p* = 0.0069], with apigenin-fed GFAP-IL6 mice having a reduced soma area (26.35 ± 1.542 μm^2^) and perimeter (21.12 ± 0.7796 μm) and a larger the number of nodes (29.87 ± 2.313) compared to control GFAP-IL6 mice (Figures 4, 5, and Supplementary Table 1). A significant “genotype” × “diet” interaction was observed for the morphometric parameters soma area [*F*(1,56) = 8.240, *p* = 0.0058] and soma perimeter [*F*(1,56) = 6.416, *p* = 0.0141].

### Apigenin Treatment Increases Microglial Arborization in GFAP-IL6 Mice

**FIGURE 6:**
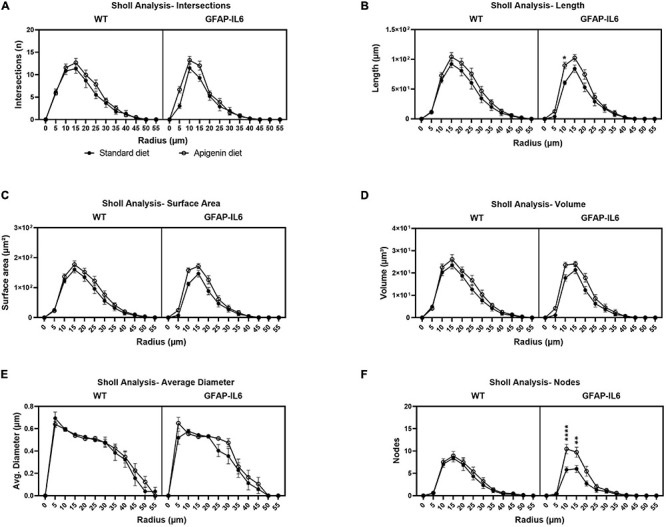
Sholl analysis used to quantify microglia arborization complexity between WT and GFAP-IL6 mice fed control and apigenin diets. Microglial arborization complexity is presented *via* microglial intersections **(A)**, length **(B)**, surface area **(C)**, volume **(D)**, average diameter **(E)**, and nodes **(F)**. In **(B)** a significant increase in process length was detected at a radius 10 μm from the soma for GFAP-IL6 apigenin-fed mice compared to GFAP-IL6 standard diet-fed mice. In **(F)** there was a significant increase in number of nodes detected at radius 10 and 15 μm for GFAP-IL6- apigenin-fed mice compared to GFAP-IL6 standard diet-fed mice. Data were analyzed using three-way ANOVA and presented as mean ± SEM. Bonferroni’s multiple comparison “diet” effect represented by asterisks (*p < 0.05, **p < 0.01, ***p < 0.001, ****p < 0.0001).

Sholl analysis was used to analyze the complexity of microglia arborization (using spheres of a given radius “*r*” centered on the soma) in the hippocampus of WT and GFAP-IL6 mice, for both control and apigenin-fed animals. The line plots and stacked charts (Figure 6 and Supplementary Figure 2) for standard diet-fed GFAP-IL6 mice show less complex microglial arborization confined to 0–25 μm radius, compared to standard diet-fed WT mice which showed interactions beyond the 25 μm radius. This is supported by significant “radius” × “genotype” effect for the number of intersections [*F*(11, 672) = 4.85, *p* < 0.0001], total process length [*F*(11, 672) = 6.26, *p* < 0.0001], surface area [*F*(11, 672) = 5.207, *p* < 0.0001], arbor volume [*F*(11, 672) = 3.627, *p* < 0.0001], average arbor diameter [*F*(11, 672) = 2.757, *p* < 0.0001], and nodes [*F*(11, 672) = 4.732, *p* < 0.0001] per concentric radii, between GFAP-IL6 standard diet-fed and WT standard diet-fed mice. Investigating this further, Bonferroni multiple comparisons show a significant decrease in total process length at 25 μm radius [WT standard diet-fed vs. GFAP-IL6 standard diet-fed; Mean difference = 31.50, ^∗^*p* = 0.0188], surface area at 25 μm [WT standard diet-fed vs. GFAP-IL6 standard diet-fed; Mean difference = 49.43, ^∗^*p* = 0.0462], and number of nodes at 20 μm radius [WT standard diet-fed vs. GFAP-IL6 standard diet-fed; Mean difference = 4.18, ^∗∗^*p* = 0.0013], for GFAP-IL6 standard diet-fed mice compared to WT standard diet-fed mice (Supplementary Figure 2).

Treatment with apigenin recovers complex microglia arborization patterns, and a significant “radius” × “diet” effect was observed with larger total length of processes [*F*(11,672) = 2.13, *p* < 0.05], surface area [*F*(11,672) = 1.956, *p* < 0.05], and nodes [*F*(11,672) = 2.544, *p* < 0.01], per 5 μm radius in apigenin fed mice compared to standard diet-fed mice. Bonferroni multiple comparisons show a significant recovery after apigenin treatment for total process length at 10 μm radius [GFAP-IL6 standard diet-fed vs. GFAP-IL6 apigenin-fed; Mean difference = −29.00, ^∗^*p* = 0.029], and the number of nodes at 10 μm [GFAP-IL6 standard diet-fed vs. GFAP-IL6 apigenin-fed; Mean difference = −4.67, ^****^p < 0.0001] and 15 μm radius [GFAP-IL6 standard diet-fed vs. GFAP-IL6 apigenin-fed; Mean difference = −3.73, ^∗∗^*p* = 0.0047], for GFAP-IL6 apigenin-fed mice compared to GFAP-IL6 standard diet-fed mice (Supplementary Figure 2). Overall, there was a “radius” × “genotype” × “diet” effect [*F*(11,672) = 1.945, *p* < 0.05] observed for the number of nodes per radii, between all cohorts.

### Hippocampal Microglial Cell Size Was Not Affected by Genotype or Diet

**FIGURE 7:**
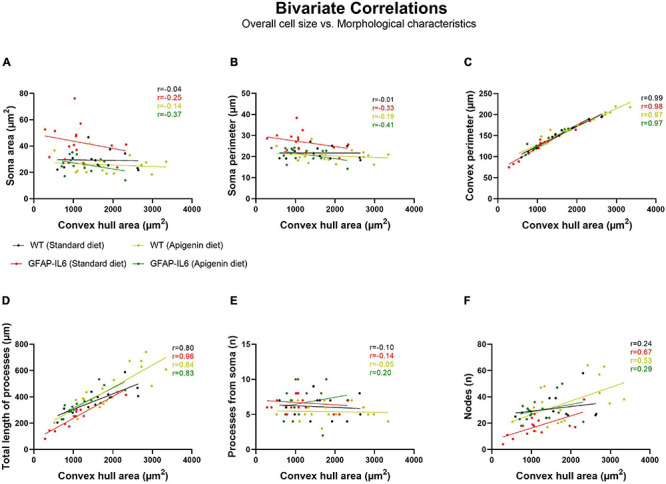
Bivariate correlations between overall microglial cell size and morphological characteristics in the hippocampus. Morphological parameters have been plotted against the convex hull area (μm^2^), including **(A)** soma area, **(B)** soma perimeter, **(C)** convex perimeter, **(D)** process length, **(E)** number of processes from soma, and **(F)** node number.

Bivariate correlation analysis of overall hippocampal microglial cell size with each morphological parameter was conducted for each genotype and treatment group. There was no association between overall microglial cell size with soma area, soma perimeter or the number of processes from the soma (Figures 7A,B,E). A significant positive association between overall microglial cell size with convex perimeter and with total length of processes from soma, was observed in all genotypes and treatment groups (Figures 7C,D). Hence, as overall microglial cell size increased, both convex perimeter and total length of process from soma increased regardless of “diet” or “genotype.” We also observed that the number of nodes increased as the overall cell size increased, regardless of “genotype” or “diet” (Figure 7F), but this positive linear association was only significantly correlated in GFAP-IL6 standard diet (^∗∗^*p* = 0.0063) and WT apigenin diet (^∗^*p* = 0.0248) groups (Table 1).

**TABLE 1:** Bivariate correlation of different morphological parameters with overall microglial cell size in the hippocampus.

		**Soma area**		**Soma perimeter**		**Convex**		**Total length of**		**Processes**			
		**(μm^2^)**		**(μm)**		**perimeter (μm)**		**processes (μm)**		**from soma**		**Nodes**	
**Cohort**	**Diet**	***R*^2^**	**Correlation**	***R*^2^**	**Correlation**	***R*^2^**	**Correlation**	***R*^2^**	**Correlation**	***R*^2^**	**Correlation**	***R*^2^**	**Correlation**
WT	Standard	0.0016	0.9005	0.0001	0.9711	0.9714	****<0.0001	0.6346	**0.0019	0.0097	0.7602	0.0576	0.4527
GFAP-IL6	Standard	0.0605	0.3770	0.1111	0.2246	0.9581	****<0.0001	0.9164	****<0.0001	0.0203	0.6123	0.4485	**0.0063
WT	Apigenin	0.0193	0.5824	0.0375	0.4412	0.9342	****<0.0001	0.7082	****<0.0001	0.0027	0.8371	0.2770	*0.0248
GFAP-IL6	Apigenin	0.1397	0.1700	0.1721	0.1242	0.9361	****<0.0001	0.6818	***0.0001	0.0401	0.4745	0.0863	0.2878

Data presented as mean ± SEM. Significant bivariate correlations indicated by asterisks (*p < 0.05, **p < 0.01, ***p < 0.001, ****p < 0.0001).

## Discussion

Neuroinflammation is hypothesized to be a major driving force behind neurodegenerative diseases. Currently, very few anti-neuroinflammatory compounds are available to treat neurodegenerative disease. Apigenin can supress inflammatory mediators in a dose-dependent manner in cell culture (Elsisi et al., 2005; Rezai-Zadeh et al., 2008) and improve cognition in rodent models (Popovic et al., 2014), making it a promising therapeutic option for the treatment of neurodegeneration. We investigated this prospect by examining the longitudinal anti-inflammatory effects of apigenin on spatial memory and microglial reactivity in the GFAP-IL6 model of neuroinflammation. We found that while chronic apigenin treatment significantly reduced several cellular measures of microglia morphology, apigenin did not improve spatial memory in GFAP-IL6 mice in the BM.

Apigenin reduced histological inflammatory markers (i.e., number of hippocampal Iba-1^+^ microglia and altered microglial morphology) in 22-month-old GFAP-IL6 mice compared with WT controls. Exploring microglia morphology could reflect altered brain physiology with a diverse impact on neuronal function. This aligns with previous studies showing apigenin treatment promotes multiple anti-inflammatory pathways, such as the prevention of NF-κB nuclear translocation (Nicholas et al., 2007) and the reduction of COX-2 expression (Lee et al., 2007), two pathways understood to control microglial function. Apigenin can also suppress CD40, TNF-α, and IL-6 production in murine microglia (Rezai-Zadeh et al., 2008). By these anti-inflammatory processes, apigenin may reduce microgliosis and ameliorate morphological microglial changes, as we have demonstrated in the GFAP-IL6 model.

Despite the reduction in inflammatory histological markers observed in the present study, apigenin treatment did not improve BM performance in aged GFAP-IL6 mice. Previous studies using a similar dose of apigenin have reported improved learning and memory following one acute apigenin treatment in young Wistar rats (Popovic et al., 2014), and after chronic apigenin treatment in the *APP×PS1* mouse model of AD (Zhao et al., 2013). However, it should be noted that these studies have utilized much younger animals than the present study (e.g., 7-month-old APP/PS1 mice) (Zhao et al., 2013), used models in which activated microglia can only be found around the amyloid plaques and the inflammatory phenotype is not generalized throughout the brain, and also employed different behavioral tasks [i.e., a passive avoidance task (Popovic et al., 2014), the MWM (Zhao et al., 2013)]. It should be noted that while both the BM and the MWM assess spatial memory, they employ different test conditions, e.g., swimming in a pool vs. finding an escape hole on a dry surface, which can affect test performance (Whishaw and Tomie, 1996; Puzzo et al., 2014). It is possible that the model used (i.e., a model of amyloid-β pathology vs. overexpression of IL-6) may impact the efficacy of apigenin to improve memory, as apigenin appears to improve cognition in *APP×PS1* mice by reducing Aβ burden, suppressing amyloidogenic process, and/or restoring the ERK/cAMP response element-binding protein/brain-derived neurotrophic factor pathway (Zhao et al., 2013). It is possible apigenin does not prevent cognitive impairment in the context of increased inflammation only, at least in this model (GFAP-IL6) and using this behavioral test (BM). This is an important finding for the field, demonstrating that apigenin may not be therapeutically beneficial for neuroinflammatory conditions alone, but may be considered therapeutically in multifactorial conditions.

We report a novel behavioral phenotype in GFAP-IL6 mice at 22 months of age. At probe, 22-month-old GFAP-IL6 mice showed no discrimination for the target hole, where discrimination was evident in WT mice at the same age, and also in GFAP-IL6 mice at 6 months of age. This finding is supported by previous research, showing age-dependent cognitive impairment in GFAP-IL6 mice in a spatial avoidance learning task, evident from 6 months of age onward (Heyser et al., 1997), and suggests a progressive decline in spatial memory in these mice. GFAP-IL6 mice may represent a model of inflammaging, whereby elevated inflammation combined with increased age causes cognitive impairment relevant to neurodegenerative disease. GFAP-IL6 mice show age-dependent reductions in cerebellar volume and PSD-95 protein levels from 14 months of age, which are not evident at 6 months (Gyengesi et al., 2019). It is possible that the age-dependent reduction in PSD-95 contributes to the impaired recall in the BM in GFAP-IL6 mice, as other manipulations which induce inflammation and reduce spatial memory (e.g., lipopolysaccharide administration) also reduce PSD-95 protein levels (Liu et al., 2018; Huang et al., 2020). Cerebellar volume loss may not contribute to spatial recall impairment in GFAP-IL6 mice, as a mouse model of AD which shows enhanced inflammation as well as spatial learning and memory deficits actually shows increased cerebellar volume (Badea et al., 2019). Also, loss of spatial memory function in aged rats is not associated with changes to cerebellar volume (Hamezah et al., 2017). Thus, it is possible lower PSD-95 protein levels may contribute to reduced recall at the probe test in 22-month-old GFAP-IL6 mice.

We detected a slower latency to find the escape hole during task acquisition in GFAP-IL6 mice at 22 months of age. While this may suggest age-dependent cognitive impairment in GFAP-IL6 mice, we also observed a trend for reduced speed during acquisition in GFAP-IL6 mice at 22 months and reduced distance traveled at probe irrespective of age, indicating some locomotor impairment in these mice, similar to that reported previously in these mice from 6 months of age onward (Gyengesi et al., 2019). It is possible that locomotor impairment may contribute to the slower acquisition latency evident in GFAP-IL6 mice at 22 months; indeed, average speed during acquisition tended to be lower in GFAP-IL6 mice at 22 months, supporting this interpretation.

However, as path length and errors during acquisition were not different between WT and GFAP-IL6 mice at 22 months, it appears GFAP-IL6 mice were slower, but equally efficient, in finding the escape hole during acquisition. This suggests that learning of the BM task may be intact in aged GFAP-IL6 mice, despite their increased primary latency. This is a potential limitation in the BM analysis of the GFAP-IL6 mouse model of neuroinflammation. Future studies should aim to assess the impact of age-dependent motor deficits on cognitive/behavioral tests in the GFAP-IL6 mice. A less physically demanding test of spatial memory should be considered in the future, in order to reduce the impact of age-dependent motor deficits on cognitive/memory measures.

The lack of quantification of apigenin in the plasma and brain of these mice is one limitation of both this study, and previous apigenin studies, as the bioavailability and brain penetration of apigenin is unknown. One consideration in using this model is that chronic microglial activation in the GFAP-IL6 mice is present from an early age, perhaps prenatally, hence it is difficult to precisely estimate the onset of microglial activation in this mouse model. An inducible IL6 promoter in the brain would be a more controlled, delicate model of chronic neuroinflammation that would guarantee normal fetal development, free from IL-6 overexpression.

## Conclusion

In conclusion, this study assessed the effects of chronic apigenin on neuroinflammation and spatial memory in GFAP-IL6 mice. Despite chronic apigenin treatment significantly reducing several measures of neuroinflammation, apigenin did not improve spatial memory recall in GFAP-IL6 mice in the BM. We also showed an age-dependent loss of spatial memory recall in GFAP-IL6. This age-dependent phenotype is consistent with previous studies that describe a loss of cognitive function (Heyser et al., 1997) with age in these mice, indicating that the GFAP-IL6 mouse may represent a suitable model for age-related neurodegenerative disease.

## Data Availability Statement

The original contributions presented in the study are included in the article/Supplementary Material, further inquiries can be directed to the corresponding author/s.

## Ethics Statement

The animal study was reviewed and approved by the Western Sydney University Animal Care and Ethics Committee (approval ID: A11393).

## Author Contributions

RC organized, analyzed, and drafted the behavioral results, and contributed significantly to drafting and editing the entire manuscript. RG analyzed and drafted the microglia morphology results and edited the manuscript. SS and CM fed and monitored the animal cohorts up to 6 months and ran the 6 months old behavioral tests. AF fed and monitored the animal cohorts from 6 to 22 months and ran the 22 months behavioral tests. HL and FU processed the brain tissue for immunohistochemistry. FU performed the immunohistochemical staining and contributed to microglia stereology counting. GM selected the animal model, co-designed the experiments, wrote the grant application to fund the study, supervised students, and edited the manuscript. TK provided the critical feedback on the manuscript. EG and GN co-designed the experiments, collected and organized the behavioral data, contributed to stereology, prepared the stereology analysis and figures, drafted and edited the manuscript, and co-supervised all students working on this project. All authors contributed to the article and approved the submitted version.

## Conflict of Interest

The authors declare that the research was conducted in the absence of any commercial or financial relationships that could be construed as a potential conflict of interest.

## Publisher’s Note

All claims expressed in this article are solely those of the authors and do not necessarily represent those of their affiliated organizations, or those of the publisher, the editors and the reviewers. Any product that may be evaluated in this article, or claim that may be made by its manufacturer, is not guaranteed or endorsed by the publisher.
