# Supplementary material for: Spatial Memory and Microglia Activation in a Mouse Model of Chronic Neuroinflammation and the Anti-inflammatory Effects of Apigenin
Source: Front Neurosci. 2021 Jul 30;15:699329. doi: 10.3389/fnins.2021.699329 (PMC8363202; doi:10.3389/fnins.2021.699329)
Supplement: Supplementary file 1 [file Data_Sheet_1.docx]

**Supplementary Material.**

**Supplementary Figure 1. Mouse body weight of WT and GFAP-IL6 mice.** Body weight of WT and GFAP-IL6 mice on standard diet vs Apigenin diet separated for sex. No significant effect of ‘diet’ or ‘genotype’ was observed.

Supplementary Figure 2. Sholl analysis used to quantify microglia arborization complexity between WT and GFAP-IL6 Standard diet-fed mice. Microglial arborization complexity is presented via microglial intersections A), length B), surface area C), volume D), average diameter E) and nodes F). Data were analyzed using three-way ANOVA and presented as mean ± SEM. Bonferroni’s multiple comparison represented by asterisks (*p < 0.05, **p < 0.01, ***p < 0.001, ****p < 0.0001).

**Supplementary Table 1.** Branched structure analysis of Iba-1^+^ microglia in the hippocampus in standard diet-fed and apigenin-fed WT and GFAP-IL6 mice at 22 months. Data presented as means ± SEM.

| Genotype | Diet | Soma area (µm^2^) | Soma perimeter  (µm) | Convex 2D area  (µm^2^) | Convex perimeter  (µm) | Total length of processes (µm) | Processes from soma | Nodes |
| --- | --- | --- | --- | --- | --- | --- | --- | --- |
| Wild type-like | Standard | 29.26 ± 2.13 | 21.72 ± 0.84 | 1645 ± 193.6 | 155.1 ± 9.23 | 381.1± 28.59 | 6.08 ± 0.48 | 31.42 ± 2.80 |
| GFAP-IL6 | Standard | 42.98 ± 3.45 | 27.04 ± 1.21 | 1135 ± 153.6 | 127.5 ± 8.32 | 259.4 ± 26.71 | 6.66 ± 0.36 | 17.53 ± 2.13 |
| Wild type-like | Apigenin | 25.75 ± 1.51 | 20.43 ± 0.70 | 1889 ± 185.5 | 166.5 ± 8.24 | 453.6 ± 37.24 | 5.38 ± 0.25 | 35.67 ± 3.64 |
| GFAP-IL6 | Apigenin | 26.35 ± 1.54 | 21.12 ± 0.77 | 1213 ± 121.1 | 136.6 ± 6.21 | 347.6 ± 22.50 | 6.66 ± 0.59 | 29.87 ± 2.31 |
